# Move or Die: the Fate of the Tax Oncoprotein of HTLV-1

**DOI:** 10.3390/v3060829

**Published:** 2011-06-15

**Authors:** Julie Lodewick, Isabelle Lamsoul, Françoise Bex

**Affiliations:** 1 Institut de Recherches Microbiologiques J-M Wiame, Université Libre de Bruxelles, Avenue E. Gryson 1, B-1070 Bruxelles, Belgium; E-Mail: jlodewic@ulb.ac.be; 2 Centre National de la Recherche Scientifique, Institut de Pharmacologie et de Biologie Structurale, 205 route de de Narbonne, Toulouse 31077, France; E-Mail: lamsoul_isabelle@hotmail.com; 3 Institut de Pharmacologie et de Biologie Structurale, Université Paul Sabatier, Université de Toulouse, Toulouse 31400, France

**Keywords:** HTLV-1, Tax, retrovirus, signal transduction, NF-κB, nuclear bodies, intracellular trafficking, posttranslational modifications, leukemia

## Abstract

The HTLV-1 Tax protein both activates viral replication and is involved in HTLV-1-mediated transformation of T lymphocytes. The transforming properties of Tax include altering the expression of select cellular genes via activation of cellular pathways and perturbation of both cell cycle control mechanisms and apoptotic signals. The recent discovery that Tax undergoes a hierarchical sequence of posttranslational modifications that control its intracellular localization provides provocative insights into the mechanisms regulating Tax transcriptional and transforming activities.

## 1. Introduction

The multipotent HTLV-1 Tax oncoprotein acts on a plethora of viral and cellular processes by interfering with cellular activation pathways and cell cycle controls. Tax can complex with more than 100 cellular proteins belonging to various functional groups [1], up- or down-regulates a number of cellular genes (about 300 out of 2000 genes examined by cDNA microarrays [2]) and interferes with the stability and activity of numerous cellular effectors. Tax potently activates the expression of all HTLV-1 genes by interacting with members of the ATF/CREB (Activating Transcription factor/cyclic AMP Response Element Binding protein) family of transcription factors. This interaction increases the dimerization and affinity of binding of ATF/CREB proteins to the Tax-responsive elements (TRE) present in the HTLV-1 5′ long terminal repeat (LTR) [3–9]. The homologous transcriptional coactivators CBP and p300 are then recruited to the assembled transcription complexes through direct interaction with Tax, leading to nucleosomal histone acetylation and transcriptional activation [10–14].

The activities of Tax are associated with the accumulation of genetic and epigenetic alterations, as well as the emergence of HTLV-1 transformed T cells and adult T-cell leukemia/lymphoma (ATL/L) that occurs in 2 to 4% of infected patients after decades of asymptomatic infection [15,16]. Some of the specific activities are as follows. First, Tax perturbs cell cycle progression by acting on cellular regulatory effectors involved in the passage through cell cycle checkpoints [17–20]. Second, Tax affects mechanisms involved in the DNA damage response and apoptosis pathways [15,21]. Third, Tax activates the expression of specific cellular genes involved in proliferation and differentiation of T lymphocytes via activation of the NF-κB pathway. Tax physically interacts with IKKγ/NEMO, the regulatory subunit of the IκB kinase (IKK) complex, resulting in activation of the IKKα and IKKβ catalytic subunits of the complex, followed by phosphorylation, polyubiquitination and degradation of the NF-κB inhibitors (IκB). NF-κB heterodimers p50/RelA (also designated as p65) are released from cytoplasmic sequestration by the IκB inhibitors and the active form of p50/RelA accumulates in the nucleus for activation of NF-κB controlled promoters [22–28].

## 2. Tax Intracellular Distribution and Functional Structure

**Figure 1.**
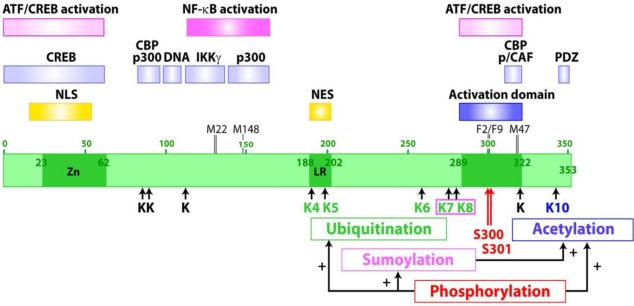
Map of the Tax oncoprotein of HTLV-1. The domains involved in transcriptional activation, in formation of complexes with relevant cellular factors, as well as posttranslational modifications and their crosstalk (arrows marked with a + sign), are shown. NLS, nuclear localization signal; NES, nuclear export signal; Zn, zinc interacting domain; LR, leucine-rich sequence.

Tax is predominantly a nuclear protein. However, cell fractionation and light microscopy studies have revealed its presence in the cytoplasm of both HTLV-1-infected T lymphocytes and a variety of cell lines transducted with Tax expression vectors. A nuclear localization signal (NLS) is present at the amino-terminus (amino acids 18 to 52) [29], overlapping a Zn interacting domain (amino acids 23 to 62) [30]. A nuclear export signal (NES) was also identified in the central domain of Tax (amino acids 188 to 202), which coincides with a leucine-rich (LR) sequence. However, the function of the NES is hidden in the context of the full-length protein [31], suggesting that modification of Tax and/or interaction with partners may be required to unmask the NES (Figure 1). When Tax is overexpressed, the distribution is heterogeneous. Cells may have Tax in either the nucleus, the cytoplasm, or at both sites [32]. In the nucleus, the striking punctate distribution of Tax was first identified as Tax speckled structures (TSS) by light microscopy [33]. After further characterization of the morphology of these nuclear structures by electron microscopy, they were re-named Tax nuclear bodies (Tax NBs) [34]. The distribution can be either diffuse or punctate throughout the whole cytoplasm or concentrated in structures associated with the Golgi apparatus. The distribution pattern depends on the cell line tested, but discrete foci in both the cytoplasm and the nucleus, are observed most frequently.

Domains involved in Tax functions have been identified by mutational analyses (Figure 1). Domains for Tax interaction with CREB [35,36] and with transcriptional coactivators CBP and p300 [12,37] as well as a domain for Tax contact with DNA [38] are at the amino-terminus. Domains important for transcriptional activation (amino acids 289 to 322) [39] and for interaction of Tax with CBP and p/CAF [13,40] are in the carboxy-terminal region. These latter domains are delineated by mutant M47 (L319R, L320S), which is selectively defective for activation of viral gene expression from the HTLV-1 promoter [41]. These amino- and carboxy-terminal domains regulate the formation of Tax/CREB/CBP/TRE quaternary complex involved in Tax-mediated activation of the HTLV-1 promoter via the ATF/CREB pathway [42]. The central region contains domains for the activation of cellular gene expression via the NF-κB pathway, including sites for interaction of Tax with IKKγ/NEMO [43] and with the transcriptional coactivator p300 [13]. Mutants M22 (T130A, L131S) [41] and M148 (G148V) [44], which are selectively defective for activation of gene expression via the NF-κB pathway, characterize the functional role of this central region. The amino-terminal zinc binding domain and three sequences localized in this central domain (DD1 127-146, DD2 181-194, DD3 213-228) control Tax dimer formation. Dimerization is critical for many functions of Tax, including assembly of the transcription complexes at the HTLV-1 promoter and the transport of Tax to the nucleus [7,45–47]. The carboxy-terminus of Tax ends with a PDZ (PSD95/Dlg/ZO-1) binding domain involved in the potent association of Tax with proteins containing PDZ domains such as hDLG (human Disc Large) [48]. The main features of Tax structure and function are shown in Figure 1.

## 3. Tax Posttranslational Modifications

Tax is modified by at least five posttranslational modifications that affect the carboxy-terminal half of the protein, including mono- and polyubiquitination, polysumoylation, proline isomerization, acetylation and phosphorylation (Figure 1). A hierarchical sequence of posttranslational modifications controls Tax intracellular localization and transcriptional activities via crosstalk.

### 3.1. Ubiquitination of Tax

The sequence of Tax includes 10 lysine residues. The five lysine residues located in the central domain (K4 to K8) are targets for polyubiquitination. However, massive degradation of Tax does not follow [32,49–51], even though Tax can bind to the nuclear proteasome [52]. The modification involving the Ubc13 ubiquitin-conjugating enzyme creates branching at lysine K63 of ubiquitin and is likely critical for both Tax interaction with IKKγ/NEMO and Tax activation of the NF-κB pathway [53]. The stable K63-branched polyubiquitinated Tax molecules are predominantly detected in cytoplasmic structures associated with the Golgi apparatus [32,54]. Polyubiquitination of Tax is essential for the assembly of active IKK complexes and RelA nuclear translocation. However, mutant K7-8R, with the two lysine residues K7 and K8 substituted by arginines, is ubiquitinated, assembles active IKK complexes and induces the translocation of RelA to the nucleus, but is defective for NF-κB activation. Thus, ubiquitination is necessary but not sufficient for activation of the NF-κB pathway [32,51].

Tax interaction with the ubiquitin E3 ligase PDLIM2 (PDZ-LIM domain-containing protein) leads to K48-branched polyubiquitinated Tax molecules, which are targeted to the nuclear matrix for proteasomal degradation [55]. It is important to note that PDLIM2 also ubiquitinates the RelA subunit of NF-κB in the nucleus and targets it to the PML nuclear bodies for proteasomal degradation [56]. Whether the observed suppression of Tax-mediated tumorigenesis by PDLIM2 [57] is due either to Tax degradation or loss of NF-κB activation following RelA degradation, or both, is not known. Tax is also monoubiquitinated at lysine residues K7 and K8 following genotoxic and cellular stress, leading to its nuclear export in a CRM-1-dependent manner [58]. K48-linked polyubiquitinated Tax is only detected following treatment with the PS-341 proteasome inhibitor [59]. The fact that ubiquitinated Tax molecules are poorly detected in the nucleus is likely to be the result of either rapid degradation or export to the cytoplasm.

### 3.2. Sumoylation of Tax

Lysines K7 (amino acid 280) and K8 (amino acid 284) are targets for polysumoylation and overlap with lysine residues that can be ubiquitinated. The ubiquitination and sumoylation at lysines K7 and K8 are exclusive since polyubiquitinated Tax molecules are predominantly detected in Golgi-associated structures in the cytoplasm, whereas polysumoylated Tax is only detected in the Tax NBs [32]. Sumoylation is critical for the assembly of Tax NBs and the recruitment of a variety of cellular proteins into these structures. Analysis of the phenotypes of the mutants of five central lysine residues (K4 to K8) revealed that both the ubiquitinated form of Tax in the cytoplasm and the sumoylated form in the Tax NBs cooperate for activation of the NF-κB pathway. The ubiquitination and sumoylation deficient K4-8R mutant is diffusely distributed both in the cytoplasm and in the nucleus and is defective for activation of gene expression via the NF-κB pathway. When lysines K4 to K6 are reintroduced into mutant K4-8R (mutant R4-6K), ubiquitination is restored but not sumoylation. However, the same diffuse distribution as in the K4-8R mutant is observed, as well as the deficiency in activation of the NF-κB pathway. The fact that fusion of SUMO-1 to the carboxy-terminus of this mutant partly restores formation of Tax NBs and Tax transcriptional activity supports the idea that both ubiquitinated and sumoylated Tax molecules act in concert for activation of the NF-κB pathway [32,51]. In contrast to Tax-mediated activation of the NF-κB pathway, activation of HTLV-1 gene expression via the ATF/CREB pathway is favored by sumoylation, but does not require ubiquitination [32].

### 3.3. Proline Isomerization of Tax

Tax is also modified by the proline-isomerization activity of Pin1 (peptidyl prolyl cis/trans isomerize) [60,61]. Pin1 is overexpressed in HTLV-1 infected T lymphocyte cell lines, and in turn, Pin1 stabilizes Tax by suppressing its ubiquitination and proteasomal degradation. Both HTLV-1 infected T lymphocytes and Tax expressing cells have Pin1 present in complexes with Tax. Importantly, Pin1 strongly stimulates Tax-mediated cell proliferation and activation of the NF-κB pathway, an effect which correlates with increased interaction of Tax with IKKγ/NEMO. It is worth noting that both the RelA subunit of NF-κB and the tumor suppressor p53, as well as other transcription and mitosis regulators, are stabilized and activated as a result of being targeted by Pin1 [62,63]. The contribution of RelA and/or p53 isomerization by Pin1 to Tax-mediated activation of the NF-κB pathway and cellular proliferation is unknown. Pin-1 isomerizes proline residues in motifs formed by a phosphoserine or threonine followed by a proline (pS/pTP). Six S/TP motifs are conserved in the Tax amino acid sequence. Of the six S/TP motifs, mutants of S113 (S113A) and S160 (S160A) do not interact with Pin1. The serine residues do not appear to be targets for phosphorylation (see Section 3.5). Both S113A and S160A mutants display increased NF-κB activation in the presence of Pin1 [64]. Thus the definitive proof that Tax is isomerized by Pin1 relies on demonstration that the isomerized form of Tax is present in Tax expressing cells, as well as the identification of the pS/pTP motif(s) of Tax targeted by Pin1.

### 3.4. Acetylation of Tax

Tax is acetylated at lysine K10 (amino acid 346) by the acetyltransferase activity of the transcriptional coactivator p300 [65]. Mutation of lysine K10 (mutant K10R) totally abolishes Tax acetylation. Antibody specific for the acetylated form of Tax shows that this form colocalizes with p300 in the Tax NBs [66], suggesting that Tax acetylation occurs in the Tax NBs. This is supported by the fact that sumoylation deficient mutants, which do not assemble Tax NBs, have a markedly reduced ability to be acetylated. Moreover, the phosphorylation deficient F2 mutant that is unable to migrate to the nucleus is also deficient for acetylation (Section 3.5). The improved Tax acetylation that is achieved by coexpression of Tax with p300 favors NF-κB activation by wild type Tax, but not by the acetylation deficient K10R mutant. Remarkably, stimulation of Tax-mediated NF-κB activity was dependent on integration of the reporter into the chromatin. Whether p300 acetylation of Tax, together with nucleosomal histones in promoter-bound transcription complexes, favors initiation of transcription is unknown. Thus, 6 of the 10 lysine residues of Tax are targets for posttranslational modification and these modifications control Tax transcriptional activities.

### 3.5. Phosphorylation of Tax

**Figure 2.**
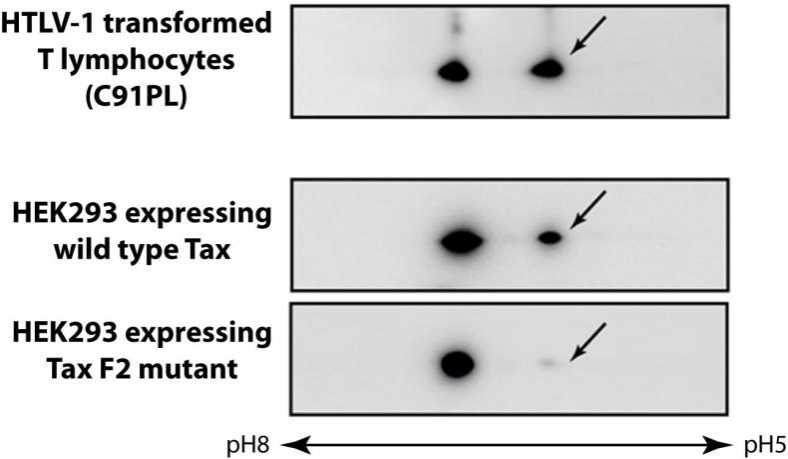
Tax is a phosphoprotein. Extracts of HTLV-1 infected T lymphocyte cell line C91PL or HEK293 cells expressing either wild type Tax or the phosphorylation defective F2 mutant were separated by two dimensional gel electrophoresis and analyzed by Western blotting using a monoclonal antibody directed against Tax. The arrows point to phosphorylated forms of Tax.

The Tax mutants F2 (S300L, S301A) and F9 (S300D, S301D) were instrumental in determining that S300 and/or S301 in the Tax activation domain were targets for phosphorylation [67,68]. In Western blots of two dimensional gels of Tax expressing cells, a Tax species with isoelectric pH more acidic than the non-modified form was detected by the anti-Tax monoclonal antibody in both HTLV-1 infected T lymphocytes and wild type Tax expressing cells, but not in cells expressing the F2 mutant (arrow, Figure 2). Metabolic labeling of HEK293 cells expressing Tax with ^32^P orthophosphate indicated that the acidic form of Tax is phosphorylated [66].

The phosphorylation deficient F2 mutant is predominantly localized in the cytoplasm and is unable to activate gene expression via either the ATF/CREB or NF-κB pathways. Replacement of the critical serine residues S300 and S301 by phosphorylation mimetic aspartic acids (F9 mutant) partly restores Tax nuclear localization and transcriptional activities. Thus, the phosphorylation at serine residues 300/301 that likely occurs in the cytoplasm is critical for Tax migration to the nucleus. As expected, the F2 mutant is deficient for sumoylation and acetylation, which occur in the nucleus. In addition, mutant F2 is deficient for ubiquitination, suggesting that phosphorylation of Tax is a prerequisite for Tax ubiquitination in the cytoplasm [65]. Importantly, the phosphomimetic F9 mutant is acetylated [65] and also recovers polyubiquitination and polysumoylation [66], supporting the conclusion that phosphorylation is a prerequisite for Tax acetylation, ubiquitination and sumoylation. The phosphorylation deficiency of mutant F2 does not preclude the phosphorylation of other Tax amino acid residues. The F2 mutations might prevent other phosphorylation events, by virtue of the fact that F2 mutant is not targeted to the nucleus and would not encounter the needed kinase(s) that reside in the nucleus. Four novel sites for phosphorylation (T48, T184, T215 and S336) have been identified by liquid chromatography tandem mass spectrometry of affinity purified Tax. The mutations of some of these residues have consequences for Tax transcriptional activities [68]. In addition, kinase CK2 phosphorylates S336, S344 and T351 *in vitro* [69].

## 4. Distribution of Tax in the Nucleus

### 4.1. Structure of the Tax Nuclear Bodies

**Figure 3.**
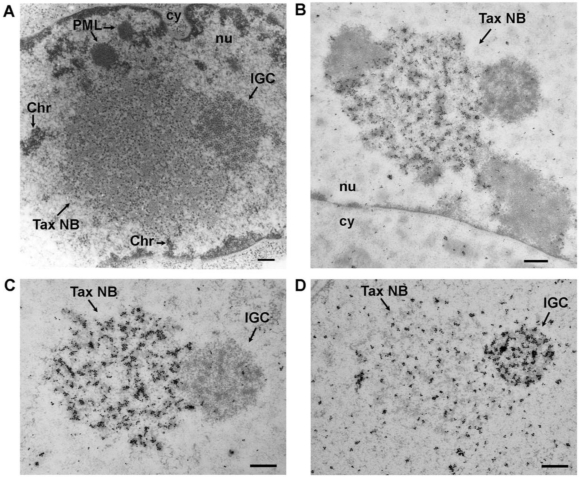
Structure of the Tax nuclear bodies. (**A**) Thin section electron microscopy of Epon embedded Tax expressing BHK21 cells. (**B**) and (**C**) Immunogold labeling with an anti-Tax rabbit polyclonal antibody followed by a secondary antibody conjugated to 15 nm colloidal gold of Lowicryl embedded Tax expressing BHK21 cells. (**D**) Immunogold labeling with an anti-Sm monoclonal antibody and a secondary antibody conjugated to 15 nm colloidal gold. Tax NB, Tax nuclear body; IGC, interchromatin granule cluster; Chr, chromatin; nu, nucleus; cy, cytoplasm. Bar, 0.5 μm.

In the nucleus, Tax occurs in discrete nuclear foci designated as Tax speckled structures (TSS) [33]. These foci, first observed by immunofluorescence staining and confocal microscopy, were then characterized by thin section electron microscopy and immunogold labeling [34,70], revealing that the Tax foci were not aggregates, but prominent nuclear bodies. Unique spherical structures of 2 to 4 μm in diameter with a well-defined architecture of electron dense 20 nm granules aligned along fibers was observed in Tax expressing cells (Figure 3A). These structures were specifically labeled with immunogold using an antibody directed against Tax (Figure 3B and C) and subsequently named Tax nuclear bodies (Tax NBs). The Tax NBs are localized in the interchromatin space and tightly associated with the nuclear matrix. Contacts with loops of decondensed chromatin occur at the surface, but DNA is not present in the core of the structure (Figure 3A, Chr), as evidenced by using a highly sensitive technique for the detection of DNA by electron microscopy [70].

Not only chromatin, but also native nuclear structures are in close spatial contact with Tax NBs. Clusters of interchromatin granules (IGC) also known as nuclear (or Sm) speckles are composed of a series of small nuclear RNPs (snRNPs) containing particles measuring 20–25 nm in diameter. They also contain the non-snRNP splicing factor SC35, a member of the SR family of splicing factors. These structures are often observed close to highly active transcription sites. They are involved in the storage and the redistribution of pre-mRNA splicing factors to sites of active transcription, but they do not contain nascent RNA and they are not directly involved in transcription [71–73]. Evidence for IGC being found at the boundaries of the Tax NBs [34] was obtained by thin section electron microscopy of Tax expressing cells embedded in Epon resin, which best preserves the subcellular architecture (Figure 3A) and by immunogold labeling using anti-Sm antibody specific for a common epitope of the core snRNPs components (Figure 3D). Sm labeled splicing complexes were not only concentrated in the peripheral IGC structures, but also in the Tax NB, although with a less dense distribution (Figure 3D). The presence of Tax and Sm in the Tax NB and the concentration of Sm, but not Tax, in the surrounding IGCs was confirmed by dual immunogold labeling with anti-Tax and anti-Sm antibodies [34]. Light microscopy indicated that another IGC component, SC35, also colocalizes with Tax in the Tax NBs [33,34]. The tight association of Tax NBs and IGCs and the presence of the Sm and SC35 components in both structures render these structures indistinguishable at the fluorescence microscope level. These observations strongly suggest that Tax does not target native IGCs but assembles unique nuclear bodies into which splicing complexes issued from IGCs are recruited.

**Figure 4.**
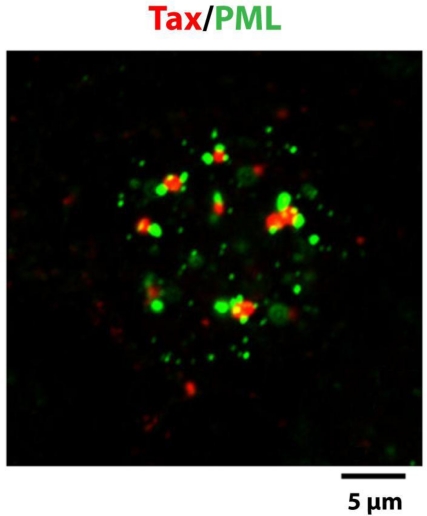
The Tax NBs and the PML NBs are distinct and adjacent entities. Immunofluorescent staining of the nucleus of a Hep2 cell expressing Tax with an anti-Tax monoclonal antibody (red) and an anti-PML rabbit polyclonal serum (green).

PML nuclear bodies (PML NBs) are native nuclear structures that are frequently found at the boundaries of the Tax NBs, both by light and electron microscopy [34,74]. The promyelocytic leukemia protein (PML) is the organizer of the PML NBs that are spheres of 0.2 to 0.5 μm in diameter and can display various morphologies. The morphologies include a light halo containing PML surrounding a dense microgranular inner core [75]. Figures 3A and 4 indicate that Tax NBs and PML NBs are distinct entities and that one or more PML NBs are often found in direct contact with the surface of the Tax NBs. The restricted interchromatin space can not by itself explain the frequent observation that PML NBs are adjacent to Tax NBs since Tax NBs are not observed in contact with other nuclear domains, such as Cajal bodies (also designated as coiled bodies) [34]. PML is conjugated to SUMO and also contains a domain known as SUMO interaction domain (SIM) for non covalent interaction with SUMO. Both of these features are critical for PML NB structural and functional integrity [76,77]. SUMO/SIM interaction is likely to be the basis for recruitment of partners, many of which are sumoylated and/or contain a SIM themselves [78,79]. Live cell imaging indicated that the association of partners with PML NBs is a dynamic process, with exchange rates in and out of the NBs specific for each partner [80]. Whether the contact between Tax and PML NBs is involved in the exchange of proteins between the two structures will be discussed in Section 4.4.

### 4.2. Composition of Tax Nuclear Bodies

More than 20 cellular proteins have been identified in the Tax NBs by light microscopy, either at the endogenous level or when overexpressed. These proteins belong to various functional categories including transcription, pre-mRNA splicing, DNA damage response, protein modification and nucleocytoplasmic transport (Table 1). For transcription, the hyperphosphorylated form of the large subunit of RNA polymerase II (RNA pol IIO) involved in elongation of mRNA transcripts has been identified in the Tax NBs. Microinjection of Alexa 488-fluorescent UTP and fluorescence *in situ* hybridization (FISH) indicated that nascent RNA, as well as RNA transcribed from a gene specifically activated by Tax via the NF-κB pathway, were present in the Tax NBs [34,70].

Besides RNA pol IIO and components of the splicing complexes Sm and SC35 (Section 4.1), the two subunits of NF-κB, p50 and RelA, are present in the Tax NBs. The active subunit of the NF-κB complex RelA is sequestered in the cytoplasm of resting cells by a family of inhibitors of NF-κB (IκB). Expression of Tax mimics cell activation. IκBα degradation is induced by Tax-mediated activation of the IKK complexes in the cytoplasm, resulting in the migration of RelA to the nucleus. In cells that do not express Tax, endogenous RelA is only detected in the cytoplasm. The expression of Tax reduces the endogenous level of RelA in the cytoplasm along with concentration of RelA in the Tax NBs. The assembly of Tax NBs that include RelA coincides with activation of expression of a NF-κB controlled promoter and is likely functionally related to Tax-mediated activation of the NF-κB pathway [34]. Interestingly, the regulatory subunit of the IKK complexes, IKKγ/NEMO, is detected at the endogenous and exogenous levels in the nucleus and expression of Tax leads to its concentration in the Tax NBs [32,81,82]. Overexpression of Tax also results in the migration of IKKγ/NEMO to the cytoplasm, where it colocalizes with Tax and several components of the NF-κB pathway (RelA, TAB2 and TAX1-BP1) [81] (discussed in Section 5).

Cellular proteins participating in activation of gene expression from the HTLV-1 promoter via the ATF/CREB pathway also concentrate in the Tax NBs. These include a member of the ATF/CREB family of transcription factors, ATF-1, and the transcriptional coactivators CBP and p300 [13]. Interactions have been detected by CHIP (Chromatin Immunoprecipitation) assays in transcription complexes associated with the HTLV-1 promoter. Inclusion of the transcriptional coactivators CBP and p300 in the complexes determines increased nucleosomal histone acetylation [14]. In addition, overexpressed transcriptional corepressor SMRT (silencing mediator of retinoic and thyroid hormone receptors) specifically stimulates Tax-mediated activation of the HTLV-1 promoter and colocalizes with Tax in the Tax NBs [74].

Several reports suggest that Tax induces genomic instability either directly by its ability to induce reactive oxygen species [94], via perturbation of cell cycle checkpoints [95] or inhibition of the DNA damage response [91,96–98]. Tax attenuates ATM kinase activity and reduces association of the damage response factor MDC1 with repair foci [99]. Expression of Tax also results in activation of the checkpoint kinase ChK2, presumably via complex formation with Tax [93]. ChK2 is recruited to Tax NBs, along with other damage repair effectors 53BP1, BRCA1, DNA-PKcs and MDC1 [91,100]. However, repair foci do not overlap with Tax NBs as indicated by the absence Nbs1, a marker of repair foci, in the Tax NBs. The authors propose that Tax sequesters MDC1 and other DNA damage repair effectors in Tax NBs, leading to their suboptimal recruitment to repair foci [91].

Several protein modification enzymes, including the acetyltransferases CBP and p300 that acetylate histones as well as Tax, kinases CDK8 and ChK2, the SUMO E2 conjugating enzyme Ubc9 and SUMO, but not ubiquitin, are components of the Tax NBs. Recently, Ubc9 was proposed to be involved in sumoylation of IKKγ/NEMO within the Tax NBs [82]. Finally, two nucleocytoplasmic export receptors, CRM-1 [70] and calreticulin [81], were detected in the Tax NBs. Expression of Tax determines partial redistribution of calreticulin and IKKγ/NEMO to the cytoplasm (discussed in Section 6).

### 4.3. Morphogenesis of the Tax Nuclear Bodies

Polysumoylation of Tax is a critical determinant for the formation of fully functional Tax NBs [32,51,59]. Mutants deficient for sumoylation (K4-8R and R4-6K) do not assemble Tax NBs and do not activate the NF-κB pathway. In contrast, mutants with restored sumoylation by reintroduction of lysines K7 and K8 on the background of mutant K4-8R (R7-8K) or by fusion of SUMO to the sumoylation deficient R4-6K mutant (R4-6K-SUMO) assemble Tax NBs and are transcriptionally active [32,51]. A recent report using deletion scanning mutants of Tax suggests that a sequence spanning amino acid residues 50 to 75 is a determinant for Tax concentration in speckled structures that contain splicing factor SC35 [45]. However, the deleted Tax Δ50-99 and truncated Tax 1–75 mutants used to delineate the 50–75 determinant are transcriptionally inactive and their sumoylation status was not tested. Moreover, Tax and SC35-containing nuclear structures that were formed after rescue by complementation were not tested for transcriptional activity. Consequently the speckled structures formed by these mutants might not be mature Tax NBs containing the full set of cellular factors involved in transcription, splicing and DNA damage response. Coimmunoprecipitation studies indicated that Tax is associated in complexes with SC35, but the domain for association has not been identified [33]. From these observations, we hypothesize that after entry in the nucleus, Tax associates with splicing complexes by an auto assembly process. During this step, Tax recruitment of SC35 from IGC would involve the Tax 50–75 amino acid sequence. Such nuclear aggregates of Tax and splicing factors have been observed at the ultrastructural level, early after initiation of Tax expression [90]. Polysumoylation of Tax would then enable the recruitment of additional cellular proteins, possibly containing SUMO-interacting motifs (SIM) by their interaction with the Tax sumoylated branches, to form fully mature Tax NBs. Arguments in favor of this hypothesis are that SUMO conjugation to target proteins significantly changes their binding properties [101] and that intermolecular interaction between branched SUMO molecules and SIM likely underlies NB biogenesis [79,102,103]. Whether Tax recruits specific cellular proteins in the Tax NBs via SUMO/SIM interaction has not been tested.

### 4.4. Function of the Tax Nuclear Bodies

There is now substantial evidence that the eukaryotic nucleus consists of highly organized structures. The chromosome territories and the nucleoli are surrounded by a dense population of nuclear speckles and bodies dispersed in the interchromatin space. The multiple functions of these interchromatin domains are emerging. Some of them, including PML NBs, are implicated in key cellular processes, such as transcription, senescence, apoptosis and response to DNA damages. They have also been implicated in the sequestration of cellular proteins away from their sites of action. Moreover, the functions of these domains may be controlled by the trafficking of specific components in and out of the structures [104–106].

**Figure 5.**
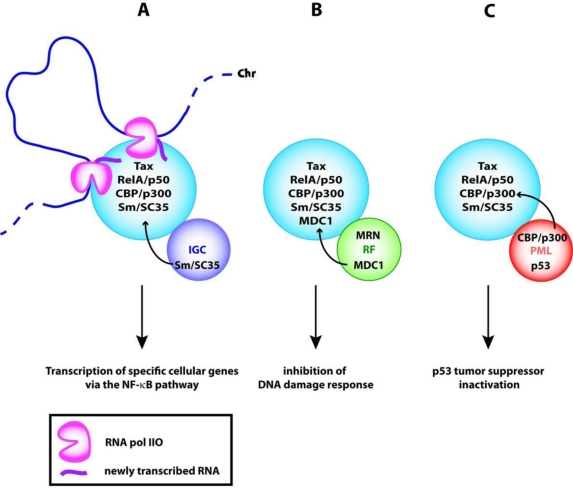
Potential functions of Tax NBs (**A**) in transcription (**B**) in sequestration of DNA repair effectors and (**C**) in sequestration of modification enzymes involved in p53 activation. IGC, interchromatin clusters; RF, repair foci; PML, PML NBs; Chr, chromatin; MRN, Mre11/Rad50/Nbs1 damage response complex.

The composition and the morphology of the Tax NBs detailed in Section 4 strongly suggest that these structures are transcription domains involved in Tax-mediated activation of gene expression. The functional role of Tax NBs in transcription is described in Figure 5A. Transcription complexes formed of RNA pol IIO, p50/RelA or ATF1, CBP and p300 would be assembled by Tax at promoters of genes present at the boundaries of the structures. The recruitment of splicing complexes (Sm, SC35) from IGCs would enable processing of pre-mRNA in the Tax NBs for their release and export to the cytoplasm. It is interesting to note that Rex, the HTLV-1 regulatory protein involved in the nuclear export of unspliced or partly spliced viral mRNA, is present in punctuate foci adjacent to the Tax NBs [70]. Thus, Tax NBs might be involved in transcription of both the HTLV-1 provirus and specific cellular genes. The proof of this hypothesis requires the identification of the genes that are in contact with the surface of the Tax NBs.

Tax NBs might also be involved in the sequestration of both DNA repair effectors and enzymes involved in transcriptional activation of the tumor suppressor p53. This is supported by the observation that Tax NBs contain MDC1 and prevent its recruitment to repair foci [91] (Figure 5B). Delocalization of the transcriptional coactivators CBP and p300 from PML NBs to the Tax NBs could explain in part how Tax functionally inactivates p53 (Figures 5C). A large body of evidence indicates that upon genotoxic stress and oncogenic transformation, the tumor suppressor p53 is recruited to PML NBs for modification by phosphorylation and acetylation. These modifications occur in PML NBs following the recruitment of homeodomain interacting protein kinase 2 (HIPK2) for phosphorylation of serine residue S46 and checkpoint kinase 2 (ChK2) for phosphorylation of S20. Moreover, the acetyltransferases CBP and p300 are dynamic components of PML NBs [107] and acetylate p53 at lysine residue K382. The subsequent stabilization and activation of p53 transcriptional activity drive senescence and apoptosis in response to genotoxic stress [108–118].

**Figure 6.**
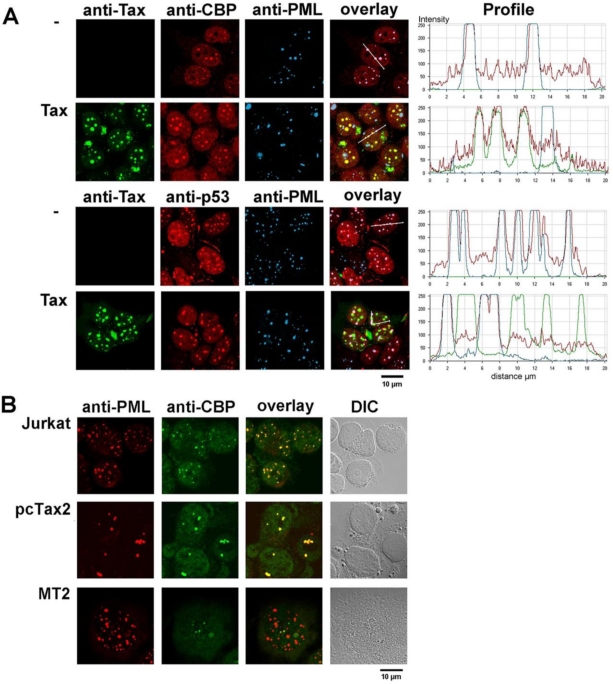
The acetyltransferase CBP is delocalized from the PML NBs to the Tax NBs. (**A**) Triple immunofluorescence staining and confocal microscopy of HEK293 cells both with and without transfection by a Tax expression vector. Either anti-Tax, anti-CBP and anti-PML antibodies or anti-Tax, anti-p53 and anti-PML antibodies were used for the staining, as indicated. The profiles of the intensity of the fluorescence for each staining along a line drawn across the nucleus are shown. (**B**) Dual immunofluorescence staining using anti-PML and anti-CBP antibodies of either uninfected T cells (Jurkat) or IL2-dependent primary lymphocytes immortalized by transfection of a cloned HTLV-1 provirus (pcTax2 [119]) or IL2-independent HTLV-1-transformed T lymphocytes (MT2). DIC, differential interference contrast.

The triple immunofluorescence staining in Figure 6A indicates that CBP is concentrated in PML NBs, in addition to a rather diffuse nuclear distribution in non-Tax expressing HEK293 cells. Expression of Tax leads to the disappearance of CBP from PML NBs and its accumulation in Tax NBs. In contrast to the delocalization of CBP, p53 is present in PML NBs and is maintained in these structures upon Tax expression. Importantly, CBP was not detected in PML NBs of HTLV-1 transformed T lymphocytes that grow independently of IL2 (MT2). In contrast, CBP colocalized with PML of uninfected T cells (Jurkat) or IL2-dependent primary lymphocytes immortalized by transfection of a cloned HTLV-1 provirus (pcTax2, [119]) (Figure 6B). Thus, the absence of CBP in PML NBs correlates with cellular transformation by HTLV-1.

## 5. Distribution of Tax in the Cytoplasm

Different patterns of Tax in the cytoplasm have been described. Some of these patterns include a diffuse or punctate distribution throughout the cytoplasm [32,81] or a more condensed distribution near the nuclear envelope, closely associated with the Golgi apparatus [32,54,59,120,121]. The components of the various cytoplasmic structures that contain Tax are listed in Table 2.

Tax localizes in perinuclear hot spots containing the Golgi apparatus markers Giantin and the Golgi matrix protein GM130, in Tax-expressing Jurkat cells and in HTLV-1-infected T cells [120]. Tax induces the concentration of the IKKα and IKKβ catalytic subunits of IKK in these Golgi-associated structures. This redistribution depends on Tax ubiquitination and ability to interact with IKKγ/NEMO [121]. Moreover, Tax copurifies with the Golgi-associated lipid raft microdomains marker GM1 in HTLV-1 infected T cells and Tax-expressing HEK293 cells. These lipid rafts include all three components of the IKK complexes, IKKα, IKKβ and IKKγ/NEMO, as well as Hsp90, an IKK-associated chaperone and TGFβ activating kinase (TAK1), an upstream IKK kinase [54]. However, another study suggests that the centrosome is the site for Tax physical interaction with IKK, resulting in the release of Tax-free active IKK complexes to the cytoplasm [59].

Our recent work indicates that Tax targets TAB2 (TAK1 binding protein 2) containing cytoplasmic dispersed foci, into which several other factors involved in NF-κB activation (IKKγ/NEMO, TAX1-BP1 and RelA) as well as calreticulin, are recruited. Ubiquitination deficient mutants associated with the TAB2 cytoplasmic foci but were unable to concentrate RelA in these structures [81]. Overexpression of IKKα or IKKβ with Tax in HEK293 cells led to their recruitment, along with endogenous IKKγ/NEMO, to subcellular punctate structures throughout the cytoplasm. Recruitment was independent of Tax ubiquitination. However, concentration of Tax, IKKγ/NEMO and RelA to Golgi-associated structures is strictly dependent on Tax ubiquitination [32]. This work suggests that ubiquitination of Tax is not *per se* required for the colocalization of Tax with IKKγ/NEMO in cytoplasmic foci, but might be required for the recruitment of RelA and the concentration of these complexes within Golgi-associated structures. The level of K63-branched polyubiquitination of Tax in different cellular models may explain the different patterns of Tax distribution in the cytoplasm.

## 6. Migration of Tax in and out of the Nucleus

In addition to (a) the amino-terminal NLS [29], (b) phosphorylation at serine residues 300/301 [65] and (c) dimerization [45], a direct interaction of Tax with p62 at the nuclear pore complexes (NPC) is needed for import of Tax into the nucleus. The nucleoporin p62 is involved in the nuclear entry of cargo proteins by interaction with importin β. The import of Tax into the nucleus appears to be carrier and energy-independent [123]. Since stable ubiquitinated Tax molecules are prominent in the cytoplasm, but minimal in the nucleus, either non-ubiquitinated Tax molecules are transported into the nucleus or polyubiquitinated Tax molecules in the cytoplasm are first deubiquitinated before migrating to the nucleus. Phosphorylation of Tax at serine residues 300/301 is critical for nuclear entry. However, the F9 mutant with substitutions of serine residues S300 and S301 by aspartic acid residues constitutively maintains phosphorylation mimicry and inefficiently migrates to the nucleus. This observation suggests that Tax might be not only deubiquitinated, but also dephosphorylated, during nuclear entry.

Tax contains a leucine-rich NES in its central domain. However, this NES is hidden in the context of the full-length protein and unmasked by truncation at amino acid 214 (truncated Tax 1–214) [31]. Since the truncated sequence includes the sumoylation targeted lysines K7 and K8 at amino acids 280 and 284, we hypothesized that sumoylation controls Tax residency in the nucleus and that desumoylation is a prerequisite for unraveling the NES and nucleocytoplasmic shuttling of Tax. Desumoylation would then be followed by mono-ubiquitination at lysines K7 and K8, which was observed to control nuclear export of Tax in a CRM-1 dependent manner upon cellular stress [58,124]. Interestingly, the nucleocytoplasmic export factor CRM-1 was detected endogenously in the Tax NBs [70]. Thus, desumoylation of Tax followed by its mono-ubiquitination might favor interaction of the NES with CRM-1 at the boundaries of the Tax NBs for efficient export of Tax to the cytoplasm.

Calreticulin might also be involved in Tax nucleocytoplasmic export. Calreticulin is a multifunctional calcium-buffering chaperone commonly used as a marker of the endoplasmic reticulum. Yet, calreticulin intracellular localization and functions also occur in the nucleus [125]. We have observed that overexpressed IKKγ/NEMO colocalized with calreticulin in nuclear foci. Coexpression of Tax with IKKγ/NEMO led to the partial redistribution of IKKγ/NEMO and calreticulin to the cytoplasm [81]. Our findings are consistent with the following previous reports. First, calreticulin is associated in complexes with Tax and the endogenous level of calreticulin is increased in HTLV-1-infected as compared to uninfected T cells [122]. Second, calcium has a unique role in NF-κB signaling by regulating the nuclear export of IKKγ/NEMO [126]. Third, calreticulin functions as a nuclear export factor for several nuclear receptors, including glucocorticoid (GR) and thyroid hormone receptors (TRα) [127,128]. It is thus possible that Tax follows an export pathway in which both CRM-1 and calreticulin cooperate, as suggested for the nuclear export of TRα [129]. Whether calreticulin-mediated export is involved in secretion of Tax [122,130] and/or recycling of Tax molecules for polyubiquitination and targeting to Golgi-associated structures [81] needs further investigation.

Finally, our results strongly suggest that ubiquitination controls the retention of Tax in the cytoplasm and sumoylation controls the retention in the nucleus. Fusion of SUMO to the carboxy-terminus of wild type Tax increases the fraction of 293T cells displaying Tax in nuclear bodies and the size of the Tax NBs, while reducing the fraction of cells having Tax in the cytoplasm. Inversely, either coexpression of ubiquitin with Tax or fusion of ubiquitin to its carboxy-terminus increases the fraction of cells having Tax concentrated in the cytoplasmic Golgi-associated structures. In addition, the ubiquitination and sumoylation defective K4-8R mutant is uniformly distributed in the cytoplasm and the nucleus in 293T cells [32]. These observations suggest that unmodified Tax migrates freely in and out of the nucleus. It is noteworthy to mention that coexpression of ubiquitin [49] or SUMO has a minimal impact, or even reduces, Tax transcriptional activities [66]. Therefore we propose that the correct balance between the level of sumoylation for assembly of fully functional NBs and the level of ubiquitination for the activation of the IKK complexes in the cytoplasmic Golgi-associated structures is required to ensure optimal transcriptional activity.

## 7. Tax Intracellular Trafficking and Activation of the NF-κB Pathway

The provocative title of this review “Move or die: the fate of the Tax oncoprotein of HTLV-1” refers to the evidence that various modified forms of Tax are differentially distributed in the cell and functionally cooperate for Tax activity. Any defect in Tax modification results in Tax mislocalization and correlates with a loss in Tax transcriptional and transforming activities. For instance, the phosphorylation deficient F2 mutant does not migrate to the nucleus, it is deficient for all other modifications and lacks transcriptional and transforming activities contrary to the phosphomimetic F9 mutant. Similarly, the ubiquitination and sumoylation deficient K4-8R mutant does not assemble nuclear bodies and is unable to activate the NF-κB pathway.

**Figure 7.**
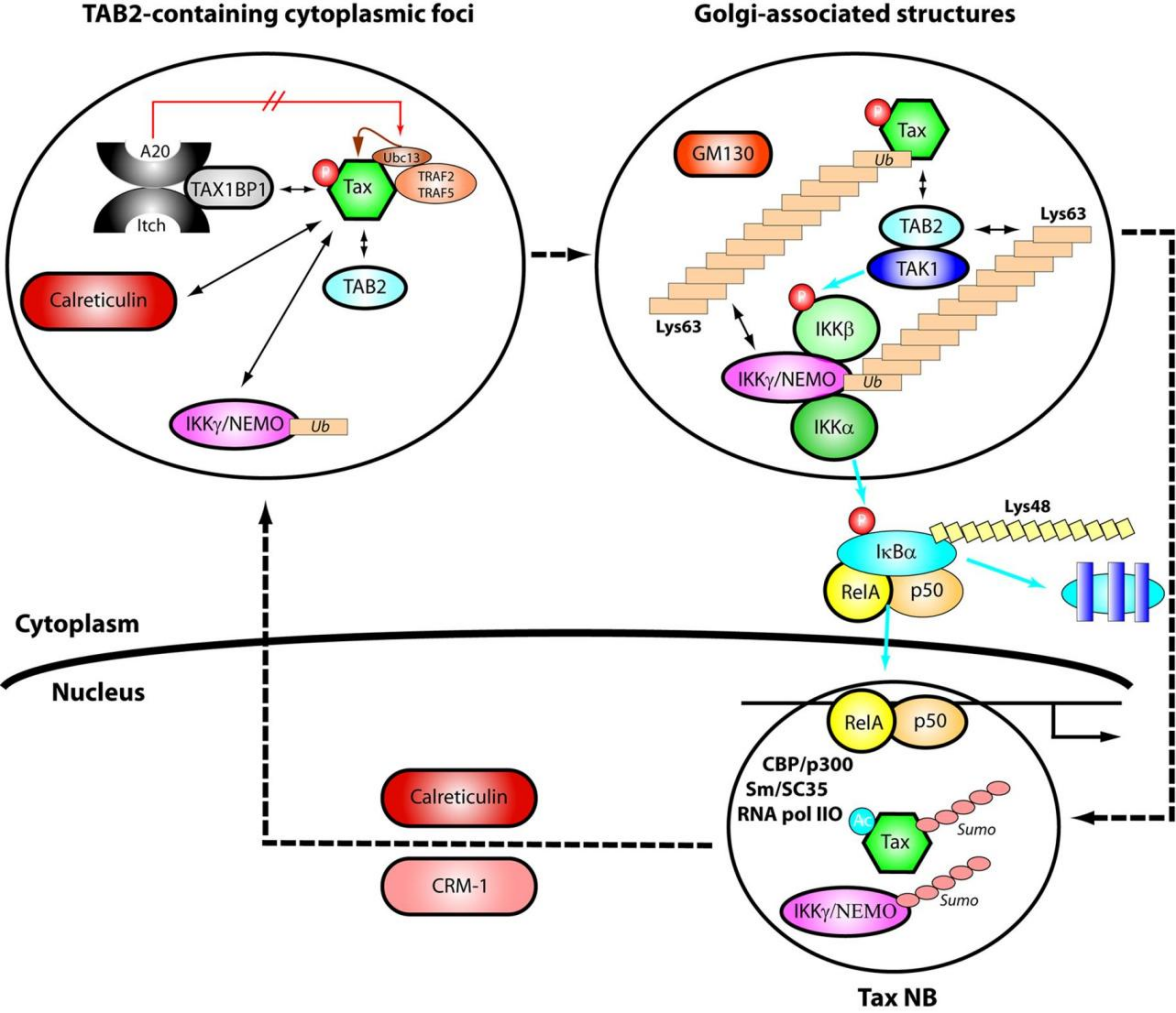
Model for the trafficking of Tax through the cytoplasm and the nucleus and its involvement in Tax-mediated activation of the NF-κB pathway. Dashed arrows denote trafficking of Tax and IKKγ/NEMO; dual headed arrows denote relevant protein-protein or protein-polyubiquitin interactions; blue arrows denote effects consecutive to NF-κB activation: phosphorylation of the IKKβ by TAK1, phosphorylation of IκBα by IKKβ, degradation of IκBα or migration of p50/RelA complexes to the nucleus. The indications Lys63 and Lys48 denote branching of ubiquitin at the ubiquitin lysine residues 63 or 48.

A model for the trafficking of Tax and cellular factors through the cytoplasm and the nucleus and their involvement in activation of the NF-κB pathway is shown in Figure 7. Newly synthesized cytoplasmic Tax is first phosphorylated at serines 300/301. Phosphorylation results in targeting of Tax to TAB2, contained within punctate cytoplasmic structures dispersed throughout the cytoplasm. Complexes of mono-ubiquitinated IKKγ/NEMO and calreticulin issued from the nucleus as a result of Tax action (see below), are recruited into these TAB2-containing cytoplasmic structures. This recruitment depends on Tax phosphorylation, but not ubiquitination. Phosphorylated Tax also recruits the ubiquitination machinery (Ubc13, TRAF2/5), resulting its K63-branched polyubiquitination. The presence of TAX1-BP1 (Tax binding protein 1) within the cytoplasmic structures may mean that these sites also host the ubiquitin editing A20/Itch/TAX1-BP1 complexes. Tax interaction with TAX1-BP1 and Ubc13 impairs formation of A20-Ubc13 complexes and subsequent degradation of Ubc13. Thus, Tax prevents Ubc13 degradation, which is essential for K63-branched polyubiquitination of Tax and persistent NF-κB activation [131,132].

Tightly associated polyubiquitinated Tax-IKKγ/NEMO complexes and TAB2 then migrate to Golgi-associated structures and recruit the IKK upstream kinase TAK1, the two catalytic subunits of IKK, IKKα and IKKβ, as well as the p50/RelA/IκBα complexes. These cytoplasmic events initiate the phosphorylation cascade leading to activation of IKK, followed by phosphorylation, K48-branched ubiquitination and degradation of IκBα and the migration of p50/RelA complexes to the nucleus. Dephosphorylation and desubiquitination of Tax might occur in the Golgi-associated structures, thus enabling comigration of Tax and p50/RelA complexes to the nucleus. Any failure in Tax ubiquitination would prevent targeting of Tax and its partners to the Golgi-associated structures, resulting in its accumulation in TAB2-containing cytoplasmic foci and reduction or loss of Tax-mediated activation of the NF-κB pathway.

In the nucleus, Tax associates with splicing factors via an auto assembly process. Tax polysumoylation determines the formation of mature Tax NBs that include transcription factors p50/RelA, CBP and p300, RNA pol IIO and the subsequent acetylation of Tax by p300. Transcription of specific NF-κB controlled genes and processing of the pre-mRNA by assembled complexes at the surface of the Tax NBs can then occur. The polysumoylated form of IKKγ/NEMO and the export factors CRM-1 and calreticulin are also recruited in the Tax NBs. Both Tax and IKKγ/NEMO are presumably desumoylated and then mono-ubiquitinated for their export out of the nucleus in a CRM-1 and in a calreticulin dependent manner. It is worth noting that upon genotoxic stress, the nuclear pool of IKKγ/NEMO is phosphorylated by the activated ATM (ataxia telangiectasia mutated) kinase and subsequently mono-ubiquitinated [133,134]. The resulting export of both IKKγ/NEMO and ATM to the cytoplasm leads to ATM dependent clustering of TAB2-TAK1 and IKK complexes for efficient NF-κB activation [135,136]. This pathway for NF-κB activation, which occurs upon genotoxic stress as part of the DNA damage response, was recently identified [133,137,138]. Whether Tax induces a cellular stress that initiates such a response has not been tested.

## 8. Conclusions

In this review we have integrated the vast amount of available information about Tax structure and function. Some of the specific questions that remain regarding the integrated view of the dynamic process of Tax cycling through the cells are as follows.

- Is SUMO/SIM interaction the basis for recruitment of Tax partners to the Tax NBs?
- Do the HTLV-1 provirus and specific cellular genes contact the surface of the Tax NBs in the course of viral and cellular transcription?
- What is the role of IKKγ/NEMO in the Tax NBs?
- How does the crosstalk between Tax NBs and PML NBs impact on prevention of apoptosis by Tax?
- Does calreticulin participate in Tax and IKKγ/NEMO export from the nucleus?
- How does proline isomerization by Pin1 and polyubiquitination by PDLIM2 affect Tax transcriptional and transforming activities?
- Which specific events occur in the cytoplasmic foci that contain Tax, TAB2, TAX1-BP1, IKKγ/NEMO and calreticulin?
- How does assembly of Tax/IKK complexes in the Golgi-associated structures lead to IKK activation?
- Is Tax accumulation in the Golgi involved in its transport to the nucleus?
- Must Tax be demodified in the process of nuclear entry?
- Which kinases phosphorylate Tax in the cytoplasm and in the nucleus?
- A question of central importance is whether Tax-induced genotoxic stress creates a signal in the nucleus that triggers activation of the NF-κB pathway.

Clarification of the mechanisms involved in Tax transcriptional and transforming activities will be beneficial for the discovery of efficient therapies for the treatment of HTLV-1 infected patients at risk for developing adult T cell leukemia.

## Figures and Tables

**Table 1.** Composition of the Tax nuclear bodies.

	Function/comment	Methods*	References

Components detected in the Tax NBs			

Transcription			

Tax	HTLV-1 oncoprotein	LM/EM	[33,34]
Pol IIO	Hyperphosphorylated form of RNA polymerase II	LM	[34,70]
RelA	NF-κB subunit	LM/coIP	[32,34,65,81,83]
p50	NF-κB subunit	LM/coIP	[34,84]
IKKγ/NEMO	Regulatory subunit of IκB kinase/NF-κB Essential modulator	LM/coIP	[23,27,32, 43,81,82,85–87]
ATF1	Activating Transcription Factor 1, member of CREB/ATF transcription factor family	LM	[13]
CBP and p300	Homologous transcriptional coactivators, acetyltransferases	LM/coIP	[10,13,65,88,89]
SMRT	Silencing mediator for retinoic acid and thyroid hormone receptors	LM	[74]

Splicing			

Sm	Core components of snRNP	LM/EM	[34,90]
SC-35	Member of SR protein family	LM/coIP	[33,34]

DNA damage response			

DNA-PKcs	DNA-dependent protein kinase catalytic subunit	LM	[91]
BRCA1	Breast cancer 2	LM	[91]
MDC1	Mediator of DNA damage checkpoint protein 1	LM	[91]
ChK2	Checkpoint kinase 2	LM	[92]
53BP1	p53 binding protein 1	LM	[93]

Protein modification			

CBP and p300	Homologous transcriptional coactivators, acetyltransferases	LM/coIP	[10,13,65,88,89], Figure 6
Ubc9	SUMO E2 conjugating enzyme	LM	[82]
ChK2	Checkpoint kinase 2	LM	[93]
CDK8	Cyclin-dependent kinase 8	LM	[34]
SUMO-1	Small ubiquitin-like modifier	LM	[32,51]

Nucleocytoplasmic transport			

CRM-1	Chromosome region maintenance protein 1, nuclear export	LM	[70]
Calreticulin	Calcium-buffering chaperone, nuclear export of hormone receptors	LM	[81]

Nucleotides			

Nascent RNA	Nascent ribonucleic acid	LM	[70]

Components not detected in Tax NBs			

PML	Promyelocytic leukemia protein: component of PML NBs	LM	[32,34,74], Figures 3A, 4, 6
Coilin	Component of Cajal bodies	LM	[34,90]
Nbs1	Component of the repair foci	LM	[91]
Ubiquitin (Ub)	76 amino acid peptide conjugated to lysines of target proteins, either as mono-Ub or via branching at lysine K48 or K63 of Ub	LM	[32]
p53	Tumor suppressor p53	LM	Figure 6
DNA	Desoxyribonucleic acid	LM/EM	[70,90]
Rex	HTLV-1 post-transcriptional regulator	LM	[70]

Determined by light microscopy (LM), electron microscopy (EM), coimmunoprecipitation (coIP).

**Table 2.** Tax cytoplasmic foci components.

	Function/comment	Methods*	References

Golgi-associated lipid raft microdomains			

Tax	HTLV-1 oncoprotein	LM	[32,54,120,121]
IKKγ/NEMO	Regulatory subunit of IκB kinase/NF-κB Essential Modulator	LM/LRF	[32,54,121]
IKKα	Catalytic subunit α of IKK	LM/LRF	[54,121]
IKKβ	Catalytic subunit β of IKK	LM/LRF	[54,121]
Hsp90	Heat shock protein 90, IKK-associated chaperone protein	LRF	[54]
TAK1	TGFβ activating kinase, IKK-upstream kinase	LRF	[54]
GM130	Golgi matrix protein 130, marker of the Golgi apparatus	LM	[54,120,121]
Giantin	Marker of the Golgi apparatus	LM	[120]
GM1, LAT, Calveolin	Markers of lipid rafts	LM/LRF	[54]
Ubiquitin (Ub)	76 amino acid peptide conjugated to lysines of target proteins, either as mono-Ub or via branching at lysines K48 or K63 of Ub	LM	[32]

TAB2-containing cytoplasmic foci			

Tax	HTLV-1 oncoprotein	LM	[81]
RelA	NF-κB subunit	LM/coIP	[81]
Calreticulin	Calcium-buffering chaperone, nuclear export of hormone receptors	LM/coIP	[81,122]
IKKγ/NEMO	Regulatory subunit of IκB kinase/NF-κB Essential Modulator	LM/coIP	[32,81]
IKKα	Catalytic subunit α of IKK	LM	[32]
IKKβ	Catalytic subunit β of IKK	LM	[32]
TAB2	TGFβ activating kinase binding protein 2	LM	[81]
TAX1-BP1	TAX1 binding protein 1, component of the A20/Itch/TAX1-BP1 ubiquitin editing complexes	LM	[81]

Centrosome			

Tax	HTLV-1 oncoprotein	LM/coIP	[59]
γ-Tubulin	Marker of the centrosome	LM/coIP	[59]
IKKγ/NEMO	Regulatory subunit of IκB kinase/NF-κB Essential Modulator	LM/coIP	[59]
IKKα	Catalytic subunit α of IKK	coIP	[59]
IKKβ	Catalytic subunit β of IKK	coIP	[59]

* Determined by light microscopy (LM), lipid raft fractionation (LRF), coimmunoprecipitation (coIP).
